# Osteoblast Attachment Compromised by High and Low Temperature of Titanium and Its Restoration by UV Photofunctionalization

**DOI:** 10.3390/ma14195493

**Published:** 2021-09-23

**Authors:** Takayuki Ikeda, Takahisa Okubo, Juri Saruta, Makoto Hirota, Hiroaki Kitajima, Naoki Yanagisawa, Takahiro Ogawa

**Affiliations:** 1 Weintraub Center for Reconstructive Biotechnology, Division of Advanced Prosthodontics, UCLA School of Dentistry, Los Angeles, CA 90090-1668, USA; okubotakahisa@gmail.com (T.O.); saruta@kdu.ac.jp (J.S.); mhirota@yokohama-cu.ac.jp (M.H.); hiroaki0315@g.ucla.edu (H.K.); togawa@dentistry.ucla.edu (T.O.); 2 Department of Complete Denture Prosthodontics, Nihon University School of Dentistry, 1-8-13 Kanda Surugadai, Chiyoda-ku, Tokyo 101-8310, Japan; 3 Department of Partial Denture Prosthodontics, Nihon University School of Dentistry, 1-8-13 Kanda Surugadai, Chiyoda-ku, Tokyo 101-8310, Japan; dena20021@g.nihon-u.ac.jp; 4 Department of Oral Science, Graduate School of Dentistry, Kanagawa Dental University, 82 Inaoka, Kanagawa, Yokosuka 238-8580, Japan; 5 Department of Oral and Maxillofacial Surgery/Orthodontics, Yokohama City University Medical Center, 4-57 Urafune-cho, Kanagawa, Yokohama 232-0024, Japan

**Keywords:** titanium, initial cell attachment, temperature, UV treatment, implant

## Abstract

Titanium implants undergo temperature fluctuations during manufacturing, transport, and storage. However, it is unknown how this affects their bioactivity. Herein, we explored how storage (six months, dark conditions) and temperature fluctuations (5–50 °C) affected the bioactivity of titanium implants. Stored and fresh acid-etched titanium disks were exposed to different temperatures for 30 min under wet or dry conditions, and their hydrophilicity/hydrophobicity and bioactivity (using osteoblasts derived from rat bone marrow) were evaluated. Ultraviolet (UV) treatment was evaluated as a method of restoring the bioactivity. The fresh samples were superhydrophilic after holding at 5 or 25 °C under wet or dry conditions, and hydrophilic after holding at 50 °C. In contrast, all the stored samples were hydrophobic. For both fresh and stored samples, exposure to 5 or 50 °C reduced osteoblast attachment compared to holding at 25 °C under both wet and dry conditions. Regression analysis indicated that holding at 31 °C would maximize cell attachment (*p* < 0.05). After UV treatment, cell attachment was the same or better than that before temperature fluctuations. Overall, titanium surfaces may have lower bioactivity when the temperature fluctuates by ≥20 °C (particularly toward lower temperatures), independent of the hydrophilicity/hydrophobicity. UV treatment was effective in restoring the temperature-compromised bioactivity.

## 1. Introduction

Titanium implants are commonly used for the restoration of missing teeth and in the repair of fractured bones and joints. Titanium is highly biocompatible, thus most implants used as intraosseous anchors are made of commercially pure titanium or titanium alloys. Successful implant anchorage is dependent on the proportion of bone in direct contact with the titanium surface without soft or connective tissue intervention, which is referred to as bone–titanium integration or osseointegration [1,2,3,4,5,6,7,8]. Successful osseointegration relies on the attachment and adhesion of bone-producing cells, such as osteoblasts, osteoprogenitor cells, and stem cells, to the titanium surface [9,10,11,12,13,14,15,16]. Titanium surfaces are highly bioactive immediately after processing [17,18,19,20,21,22,23,24] and facilitate increased attachment and functionality of osteoblasts, which leads to enhanced bone formation on the surface [17,19,22,25,26,27]. A newly fabricated titanium surface is superhydrophilic with a water contact angle of 0°. However, this hydrophilicity is lost over time, along with a reduction in bioactivity and bone-forming capability [17,19,22,25,26,27]. Specifically, titanium surfaces stored for 4 weeks after processing have been reported to have a 50% loss in capability to attract osteoblasts compared to newly processed surfaces associated with an increase in hydrophobicity [17,19,22,26,27,28,29].

Implant products used in both dental and orthopedic treatments are typically sold in sterile packaging containing either air or liquid (e.g., water or saline solution) [24,29,30]. During inventory management, transportation, and distribution, these implant products are inevitably exposed to temperature fluctuations above and below room temperature (~25 °C). Storage at the peripheral user level, such as dental practices and orthopedic hospitals, is also often associated with further deviations from room temperature. It is unknown whether these temperature fluctuations affect the superhydrophilicity and bioactivity of titanium implants. If there is a negative impact of temperature fluctuations, a potential countermeasure should be explored. Recent studies revealed that ultraviolet (UV) light treatment of titanium restored its superhydrophilicity after aging/storage [19,31,32,33,34,35,36]. UV treatment has also been reported to remove surface impurities (e.g., hydrocarbons) [32,37,38,39,40], thereby enhancing the biological activity of titanium for osteoblasts and bone [15,16,19,20,25,28,31,33,34,35,40,41,42,43,44,45,46,47,48,49,50,51,52,53,54,55,56,57,58,59,60,61,62,63].

This study aimed to determine the effect of temperature deviations from room temperature on the bioactivity of titanium implant materials. Further, UV treatment was evaluated to recover any loss in superhydrophilicity due to temperature deviations. These findings provided valuable insights regarding the effect of temperature fluctuations during titanium implant manufacturing, transport, and storage on the ability of the titanium implant surface to attract cells.

## 2. Materials and Methods

### 2.1. Titanium Disk Preparation

Commercially pure titanium disks (grade 2; diameter = 20 mm; thickness = 1.0 mm) were acid-etched in 67% sulfuric acid at 120 °C for 75 s. The acid-etched titanium disks were divided into two experimental groups, namely immediate use after preparation (fresh) and storage in dark conditions (temperature = 25 °C; humidity = 58%) for six months (old). The fresh and old titanium disks were held at 5, 25, or 50 °C for 30 min in ambient atmosphere (dry conditions) or submerged in double distilled water (ddH_2_O) (wet conditions). Titanium disks can be classified into four groups, viz. old-dry, fresh-dry, old-wet, and fresh-wet. Each group contains disks exposed to three different temperatures: 5, 25, and 50 °C. Therefore, a total of 12 titanium disks are required. In addition, since the evaluation was performed using three titanium disks (*n* = 3), a total of 36 titanium disks were used in one experiment. Subsequently, 18 disks from two different groups (old-dry and fresh-dry) were UV treated by TheraBeam SuperOsseo (Ushio, Tokyo, Japan) for 12 min.

### 2.2. Osteoblast Cell Culturing

Bone marrow-derived osteoblasts were isolated from the femurs of eight-week-old male Sprague Dawley rats according to a previously reported procedure [10,11,64,65,66,67,68]. The Nihon University Animal Research Committee approved this protocol (AP18DEN019-1, approved on 31 July 2018). The osteoblasts were transferred to alpha-modified Eagle’s medium supplemented with 15% fetal bovine serum, 50 µg/mL ascorbic acid, 10 mM Na-β-glycerophosphate, 10^−8^ M dexamethasone, and antibiotic-antimycotic solution containing 10,000 units/mL penicillin G sodium, 10,000 mg/mL streptomycin sulfate, and 25 mg/mL amphotericin B. The cells were incubated in a humidified atmosphere of 95% air and 5% CO_2_ at 37 °C. After reaching 80% confluence, the cells were detached using 0.25% trypsin-1 mM EDTA-4Na solution. 

The prepared titanium disks were transferred to 12-well culture dishes, and the cultured cells were seeded onto the titanium disks at a density of 3 × 10^4^ cells/cm^2^. The cells were incubated for 30 min before initial cell attachment was evaluated.

### 2.3. Titanium Surface Hydrophilicity

The hydrophilicity of the prepared titanium disks with and without UV treatment were determined before and after cell culturing. The degree of hydrophilicity was evaluated based on the contact angle formed between the titanium surface and 10 µL ddH_2_O and the area of ddH_2_O spread, which were measured using an image analyzer (ImageJ; NIH, Bethesda, MD, USA).

### 2.4. Initial Cell Attachment

The initial attachment of osteoblasts to the prepared titanium disks was evaluated by measuring the number of cells on the surface using a tetrazolium salt (WST-1)-based colorimetric assay (Roche Applied Science, Mannheim, Germany). The amount of formazan product was measured using a multi-detection microplate reader (Synergy^TM^ HT, BioTek Instruments, Inc., Winooski, VT, USA) at a wavelength of 450 nm.

### 2.5. Statistical Analyses

The prepared titanium disks included old and fresh titanium disks that were exposed to temperatures of 5, 25, or 50 °C under either wet or dry conditions, giving the groups old–dry, fresh–dry, old–wet, and fresh–wet. The hydrophilicity and cell adhesion of three titanium disks (*n* = 3) were evaluated for each of the above preparation conditions. One-way ANOVA was performed to determine the differences between the various groups. Statistical significance was set at *p* < 0.05. Regression analysis was performed to determine the relationship between hydrophilicity and cell adhesion. Statistical analyses were performed using IBM SPSS Statistics version 20 (IBM, Armonk, NY, USA).

## 3. Results

The hydrophilicity and cell attachment of the various groups of prepared titanium disks were compared, as the hydrophilicity and ability of titanium to attract osteoblasts are believed to be strongly related. The initial cell attachment is dependent on the behavior of cells immediately after attachment, which was evaluated until 24 h after cell seeding. The number of initially attached cells has a significant effect on cell proliferation and cell behavior (e.g., cell differentiation).

**Figure 1:**
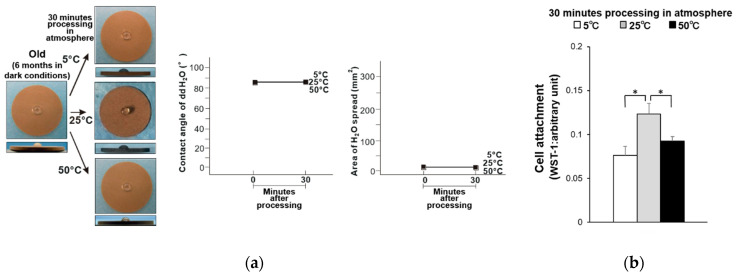
(**a**) Hydrophobicity/hydrophilicity of the old–dry titanium disk surfaces illustrated as a photograph of 10 µL ddH_2_O placed on the sample surface before and after temperature deviation (left) with the corresponding contact angle (middle) and spread area (right). (**b**) Initial attachment of osteoblasts to the old–dry titanium disk surfaces. * *p* < 0.05, statistically significant difference.

**Figure 2:**
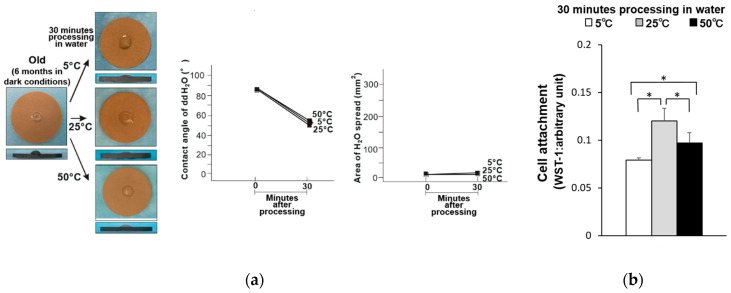
(**a**) Hydrophobicity/hydrophilicity of the old–wet titanium disk surfaces illustrated as a photograph of 10 µL ddH_2_O placed on the sample surface before and after temperature deviation (left) with the corresponding contact angle (middle) and spread area (right). (**b**) Initial attachment of osteoblasts to the old–wet titanium disk surfaces. * *p* < 0.05, statistically significant difference.

The hydrophilicity of the old-dry titanium disk surfaces was compared before and after being held at 5, 25, and 50 °C for 30 min (Figure 1a). The water contact angle was greater than 80° for all samples, and the area of water spread was small. Thus, these samples had become hydrophobic. The attachment of osteoblasts on these samples was also compared, where the titanium disks stored at 5 and 50 °C had 35% and 25% fewer attached osteoblasts compared to the titanium disk stored at 25 °C (Figure 1b). Similarly, the old-wet samples had become hydrophobic after storage at all three temperatures (Figure 2a), and the same general trend in osteoblast attachment was observed (Figure 2b).

**Figure 3:**
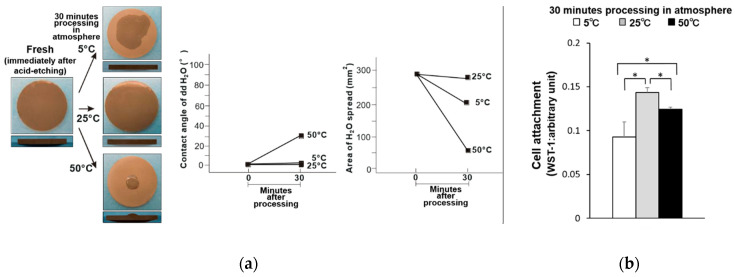
(**a**) Hydrophobicity/hydrophilicity of the fresh–dry titanium disk surfaces illustrated as a photograph of 10 µL ddH_2_O placed on the sample surface before and after temperature deviation (left) with the corresponding contact angle (middle) and spread area (right). (**b**) Initial attachment of osteoblasts to the fresh–dry titanium disk surfaces. * *p* < 0.05, statistically significant difference.

**Figure 4:**
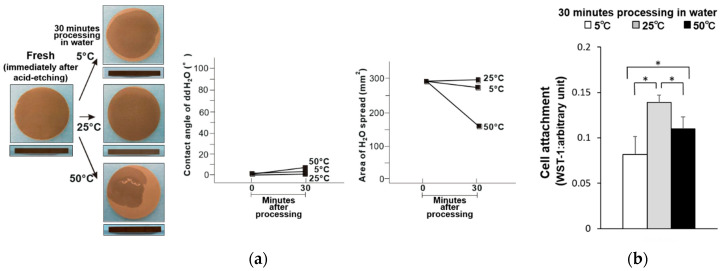
(**a**) Hydrophobicity/hydrophilicity of the fresh–wet titanium disk surfaces illustrated as a photograph of 10 µL ddH_2_O placed on the sample surface before and after temperature deviation (left) with the corresponding contact angle (middle) and spread area (right). (**b**) Initial attachment of osteoblasts to the fresh–wet titanium disk surfaces. * *p* < 0.05, statistically significant difference.

Commercialized solution-stored implants are sealed in water or saline solution immediately after surface treatment. Therefore, the titanium disks were also evaluated immediately after acid treatment (i.e., fresh titanium disk samples). The fresh–dry titanium disks became less hydrophilic when stored at 50 °C, and exhibited a contact angle of 32° (Figure 3a). However, the hydrophilicity of the fresh disks was maintained when stored at 5 and 25 °C. Significantly fewer osteoblasts were attached to the titanium disks stored at 5 and 50 °C compared to those stored at 25 °C, where the least osteoblast attachment was observed on those stored at 5 °C (Figure 3b). Similarly, the fresh–wet titanium disks stored at 5 and 25 °C remained hydrophilic, while those stored at 50 °C exhibited a partial loss of hydrophilicity and a contact angle of 7° (Figure 4a). Further, the trend in osteoblast attachment of the fresh–wet samples was similar to that of the fresh–dry samples, where less attachment occurred after storage at 5 (least) and 50 °C compared to 25 °C (Figure 4b).

**Figure 5:**
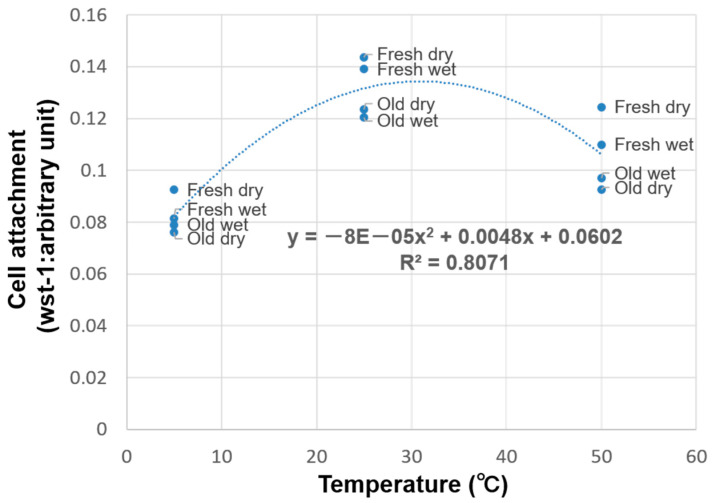
Regression analysis of titanium temperature and cell attachment using a quadratic curve (*R*^2^ = 0.81).

**Figure 6:**
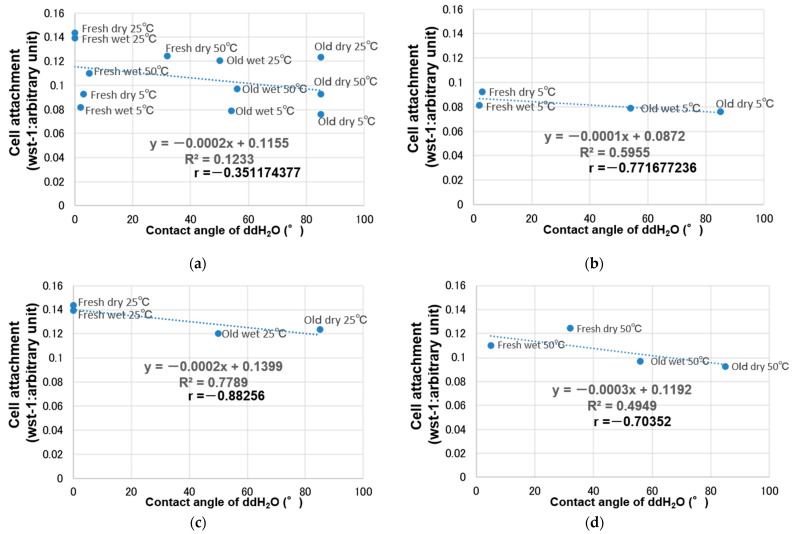
Relationship between initial cell attachment and hydrophilicity: (**a**) overall (*R* = −0.35), (**b**) at 5 °C (*R* = −0.77), (**c**) at 25 °C (*R* = −0.88), and (**d**) at 50 °C (*R* = −0.70).

The relationship between storage temperature and cell attachment was investigated using regression analysis. Cell attachment increased from 5 °C to room temperature (25 °C) and subsequently decreased from room temperature to 50 °C, thus curved regression was more suitable than linear regression. Specifically, a quadratic curve fit the relationship between storage temperature and cell attachment with a very high coefficient of determination (*R*^2^ = 0.81), and initial cell attachment peaked at 31 °C (Figure 5). Subsequently, the relationship between initial cell attachment and hydrophilicity was evaluated at each storage temperature (5, 25, and 50 °C) (Figure 6). Overall, there was no significant correlation between hydrophilicity and initial cell attachment (*R* = −0.35) (Figure 6a). However, there was some negative correlation between hydrophilicity and initial cell attachment when each storage temperature was evaluated separately (*R* = −0.77, −0.88, and −0.70 for 5, 25, and 50 °C, respectively) (Figure 6b–d).

**Figure 7:**
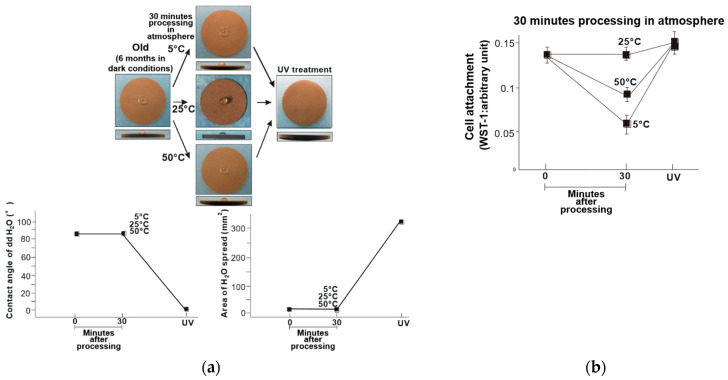
(**a**) Hydrophobicity/hydrophilicity of the UV-treated old–dry titanium disk surfaces illustrated as a photograph of 10 µL ddH_2_O placed on the sample surface before and after temperature deviation (upper middle) with the corresponding contact angle (lower left) and spread area (lower right). (**b**) Initial attachment of osteoblasts to the UV-treated old–dry titanium disk surfaces.

**Figure 8:**
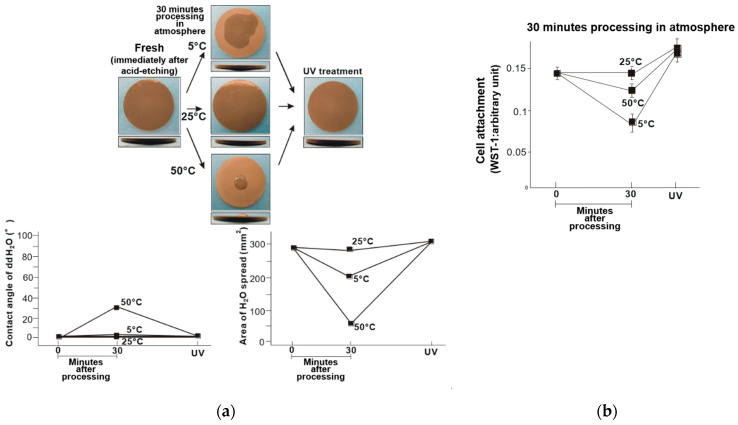
(**a**) Hydrophobicity/hydrophilicity of the UV-treated fresh–dry titanium disk surfaces illustrated as a photograph of 10 µL ddH_2_O placed on the sample surface before and after temperature deviation (upper middle) with the corresponding contact angle (lower left) and spread area (lower right). (**b**) Initial attachment of osteoblasts to the UV-treated fresh–dry titanium disk surfaces.

These findings demonstrated the effectiveness of UV treatment after titanium has been stored different temperatures. The surfaces of the titanium disks were superhydrophilic after UV treatment, regardless of storage temperature. The fresh and old titanium disks exhibited similar trends (Figure 7a and Figure 8a), and the initial cell attachment on the temperature-abused titanium disks was improved after UV treatment. Further, the number of attached cells was higher than that of the titanium disks stored at room temperature without UV treatment (Figure 7b and Figure 8b).

## 4. Discussion

Commercialized implant bodies are often roughened to enhance osseointegration [12,18,34,42,69,70,71,72,73,74,75,76,77,78,79], where acid treatment is the most common surface treatment method [4,7,9,80,81,82,83]. The surface-treated implant body is subsequently packaged dry or in solution (e.g., water or saline solution) before transportation and storage. Thus, this study investigated the effect of temperature deviations on acid-treated titanium stored under dry and wet conditions. The temperatures investigated were 5 °C, 25 °C, and 50 °C, which represent the temperatures during transportation and storage. Body temperature (37 °C) is a very important temperature for osteoblast culture and is the set temperature of the incubator. Figure 5 shows that cell attachment increases at temperatures near 37 °C. However, this temperature is not included in this study because the study was focused on temperature fluctuations during transport and storage, prior to implant placement in the oral cavity. In addition, pH is an important factor governing cell culture and function of biological cells in the oral cavity. A CO_2_ incubator was used in the present study under an established condition of temperature and CO_2_ concentration. Under the condition, culture medium is supposed to work as a buffer and maintains the pH. In fact, the pH of the culture medium was measured in this study, with or without titanium disks with different temperatures. The medium showed a slightly alkaline pH value, which was not affected by the presence of a titanium disk of room temperature and of high and low deviated temperature.

Cell attachment was investigated using osteoblasts. Osseointegration involves osteoblasts responsible for bone formation and osteoclasts responsible for bone resorption. Therefore, the effect of temperature fluctuations on osteoclasts must be elucidated. On the other hand, among the cytokines produced by osteoblasts, TGF-β1 promotes bone formation, and IL-11 promotes osteoclast formation and suppresses bone formation. In addition, TGF-β1 is also an IL-11 production promoter [84,85,86]. Therefore, osteoblasts are strongly involved in osteoclast activity. The present results certainly motivated us to pursue this line of research using osteoclasts in future projects. Pure titanium is a common material used for dental implants; therefore, we used grade 2 pure titanium as a representative target. In the medical field, there are many opportunities to use titanium alloys like Ti6A14V; therefore, further investigation is required. Bacteria are strongly involved in determining the prognosis of implants. We have studied the relationship between hydrophilicity and bacterial biofilm formation [51,87,88] and revealed the role of hydrophilicity in reducing the biofilm formation. Since the present study showed a novel relationship between the titanium temperature and the state of hydrophilicity/hydrophobicity, the effect of titanium temperature on the biofilm formation would be of an immediate future interest.

The titanium disks were hydrophilic immediately after acid treatment, and became hydrophobic and stable when stored in a dark room for 6 months and subjected to subsequent temperature fluctuations under dry and wet conditions (Figure 1 and Figure 2). Among the old–dry and old–wet samples, the best initial cell attachment was achieved after storage at 25 °C. The hydrophilicity of the acid-etched titanium disks was maintained in all fresh–dry and fresh–wet titanium disks, with the exception of the fresh–dry samples stored to 50 °C (Figure 3 and Figure 4). Similar to the old sample groups, cell attachment was significantly higher after storage at 25 °C compared to the other temperatures.

The relationship between cell attachment and temperature was best described using curved regression of the measured values, where a reasonable coefficient of determination of 0.81 was obtained (Figure 5). Further, there was no overall correlation between cell attachment and hydrophilicity (Figure 6a), but a negative correlation was noted when the samples stored at 5, 25, and 50 °C were considered separately (Figure 6b–d). This indicated that cell attachment decreased with decreasing hydrophilicity.

A titanium surface exposed to the atmosphere will become covered in a titanium oxide film. Titanium is a popular implant material because titanium oxide is stable with high corrosion resistance at room temperature, and is highly biocompatible. Therefore, temperature control is not typically considered during implant manufacturing, distribution, and storage. Further, manufacturers and users do not typically consider temperature fluctuations during titanium implant storage, as titanium has a melting point of 1812 °C and is highly resistant to corrosion. However, despite the stable surface morphology of titanium, the hydrophilicity and biological activity of titanium are lost over time [25,26]. This suggests that non-visual changes can affect the factors that are important for the functionality of the implant material.

Achieving strong osseointegration between the implant material and bone is the most important consideration during treatment, as this will ensure the long-term success of the implant. Therefore, the factors that influence osseointegration must be carefully considered, even if they seem trivial. Manufacturers have developed new surfaces for implants to achieve improved osseointegration, while other products are stored in solution immediately after titanium surface treatment to maintain hydrophilicity [89,90]. All new developments and approaches must be investigated and considered to continue improving the success rate of implant treatments.

Initial cell attachment to the implant surface will affect the subsequent cell proliferation and differentiation, and is one of the key factors in osseointegration [91]. Cell attachment is known to be compromised by the loss of titanium surface hydrophilicity, the attachment of organic substances, and the attachment of carbon [19,92]. Further, this study clearly demonstrated that temperature fluctuations can also have a significant effect, where regression analysis indicated that initial cell attachment was maximized at approximately 31 °C. Considering human body temperature, high cell attachment would be expected in the region of 31 °C instead of room temperature. However, the temperature is expected to deviate from this during implant manufacturing, transportation, and storage, where temperatures from 5 to 50 °C are commonly encountered during transport. For example, the temperature during air transportation at 33,000 feet is −56.5 °C, while the temperature inside a container during sea transportation can fluctuate from below freezing to 60 °C [93]. This exposure to extreme temperatures may limit the primary performance of an implant surface, where decreased initial cell attachment was observed in this study after storage at 5 and 50 °C in both dry and wet conditions. Further, the storage method alone does not protect against the effects of temperature deviation, thereby demonstrating that implant temperature control is important.

The hydrophilic acid-etched titanium disks became hydrophobic after 6 months of storage at all temperatures and conditions, while the titanium disks exposed to temperature fluctuations immediately after acid etching remained hydrophilic, with the exception of exposure to 50 °C in the dry state. These findings indicated that the hydrophilicity was relatively unaffected by temperature immediately after treatment, but did become hydrophobic over time. Thus, storage in solution did aid in maintaining hydrophilicity. The more hydrophilic titanium surfaces exhibited higher initial cell attachment. Previous studies have reported similar findings, regardless of titanium sample shape [41,46,55]. However, the titanium samples that were compromised due to temperature deviations exhibited no correlation between hydrophilicity and initial cell attachment.

The negative correlation between hydrophilicity and initial cell attachment at each temperature suggested that the relationship between hydrophilicity and initial cell attachment should be evaluated under constant temperature conditions. Alternatively, these results may indicate that the effect of temperature on initial cell attachment was independent of hydrophilicity. Storage in solution was more effective in maintaining hydrophilicity. However, maintaining hydrophilicity is not the only factor that plays a role in effective osseointegration, as the importance of hydrophilicity is attributed to its association with cell attachment.

The temperature fluctuations in this study mainly affected the initial cell adhesion on the titanium surface. However, it is difficult to resolve this issue during implant manufacturing and transport. Further, solution storage of the implants did not completely prevent detrimental changes. Therefore, manufacturers must consider implant temperature control. Some aircraft and tanker containers used for transportation offer temperature control functionalities, but are more expensive. A practical solution is controlling the temperature after the implant reaches the user, but this requires temperature-controlled storage equipment. Instead, UV treatment of the titanium surface can be conducted after the implant becomes hydrophobic over time, and restores the original hydrophilicity achieved immediately after surface treatment. UV treatment also removes naturally deposited hydrocarbons from the titanium surface. Overall, UV treatment can significantly enhance cell adhesion, proliferation, and alkaline phosphatase (ALP) activity, and animal studies have demonstrated that the initial osseointegration strength approximately tripled, and the bone-and-implant contact increased from between 45% and 55% to over 95% [19,25,31,32,42]. This study demonstrated that UV treatment was counteracted the effects of temperature changes, and restored the superhydrophilicity of the titanium disks exposed to all storage conditions. After the development of microrough titanium implants, implant therapy has been established as a standard measure with a high success rate. The study revealed that, albeit in vitro, the significant adverse effects of temperature fluctuations, which should be considered for future implant therapy, including the methods and conditions of transportation and storage. More importantly, UV treatment has been shown to restore the adverse effects of temperature fluctuations, implying a new function of this surface treatment that potentially contributes to further improve implant therapy.

## 5. Conclusions

This study demonstrated that the storage of titanium disks at ~20 °C above or below room temperature (25 °C) compromised the ability of the titanium surface to attract osteoblasts, where storage at low temperatures (i.e., 5 °C) had a greater detrimental effect than that observed at high temperatures (i.e., 50 °C). This reduction in bioactivity was associated with the loss of hydrophilicity of the titanium surface. UV treatment was successfully applied to restore this adverse effect of temperature deviations by regenerating hydrophilicity. These findings suggest that temperature should be carefully considered when handling titanium implant materials, although UV treatment can be effectively used to counteract the negative impacts caused by temperature abuse.

## Data Availability

The data presented in this study are available on request from the cooresponding author.

## References

[1] Zarb G.A., Schmitt A., Baker G. (1987). Tissue-Integrated Prostheses: Osseointegration Research in Toronto. Int. J. Periodontics Restor. Dent..

[2] Zarb G.A. (1985). Clinical Application of Osseointegration. An Introduction. Swed. Dent. J. Suppl..

[3] Brånemark P.I. (1983). Osseointegration and Its Experimental Background. J. Prosthet. Dent..

[4] Butz F., Ogawa T., Chang T.L., Nishimura I. (2006). Three-Dimensional Bone-Implant Integration Profiling Using Micro-Computed Tomography. Int. J. Oral Maxillofac. Implant..

[5] Ogawa T., Nishimura I. (2003). Different Bone Integration Profiles of Turned and Acid-Etched Implants Associated with Modulated Expression of Extracellular Matrix Genes. Int. J. Oral Maxillofac. Implant..

[6] Ogawa T., Nishimura I. (2006). Genes Differentially Expressed in Titanium Implant Healing. J. Dent. Res..

[7] Ogawa T., Ozawa S., Shih J.H., Ryu K.H., Sukotjo C., Yang J.M., Nishimura I. (2000). Biomechanical Evaluation of Osseous Implants Having Different Surface Topographies in Rats. J. Dent. Res..

[8] Tsukimura N., Kojima N., Kubo K., Att W., Takeuchi K., Kameyama Y., Maeda H., Ogawa T. (2008). The Effect of Superficial Chemistry of Titanium on Osteoblastic Function. J. Biomed. Mater. Res. A.

[9] Ogawa T., Sukotjo C., Nishimura I. (2002). Modulated Bone Matrix-Related Gene Expression Is Associated with Differences in Interfacial Strength of Different Implant Surface Roughness. J. Prosthodont..

[10] Nakamura H.K., Butz F., Saruwatari L., Ogawa T. (2007). A Role for Proteoglycans in Mineralized Tissue-Titanium Adhesion. J. Dent. Res..

[11] Saruwatari L., Aita H., Butz F., Nakamura H.K., Ouyang J., Yang Y., Chiou W.A., Ogawa T. (2005). Osteoblasts Generate Harder, Stiffer, and More Delamination-Resistant Mineralized Tissue on Titanium than on Polystyrene, Associated with Distinct Tissue Micro- and Ultrastructure. J. Bone Miner. Res..

[12] Takeuchi K., Saruwatari L., Nakamura H.K., Yang J.M., Ogawa T. (2005). Enhanced Intrinsic Biomechanical Properties of Osteoblastic Mineralized Tissue on Roughened Titanium Surface. J. Biomed. Mater. Res. A.

[13] Att W., Tsukimura N., Suzuki T., Ogawa T. (2007). Effect of Supramicron Roughness Characteristics Produced by 1- and 2-Step Acid Etching on the Osseointegration Capability of Titanium. Int. J. Oral Maxillofac. Implant..

[14] Nakamura H., Saruwatari L., Aita H., Takeuchi K., Ogawa T. (2005). Molecular and Biomechanical Characterization of Mineralized Tissue by Dental Pulp Cells on Titanium. J. Dent. Res..

[15] Miyauchi T., Yamada M., Yamamoto A., Iwasa F., Suzawa T., Kamijo R., Baba K., Ogawa T. (2010). The Enhanced Characteristics of Osteoblast Adhesion to Photofunctionalized Nanoscale TiO_2_ Layers on Biomaterials Surfaces. Biomaterials.

[16] Yamada M., Miyauchi T., Yamamoto A., Iwasa F., Takeuchi M., Anpo M., Sakurai K., Baba K., Ogawa T. (2010). Enhancement of Adhesion Strength and Cellular Stiffness of Osteoblasts on Mirror-Polished Titanium Surface by UV-Photofunctionalization. Acta Biomater..

[17] Hori N., Att W., Ueno T., Sato N., Yamada M., Saruwatari L., Suzuki T., Ogawa T. (2009). Age-Dependent Degradation of the Protein Adsorption Capacity of Titanium. J. Dent. Res..

[18] Hori N., Iwasa F., Ueno T., Takeuchi K., Tsukimura N., Yamada M., Hattori M., Yamamoto A., Ogawa T. (2010). Selective Cell Affinity of Biomimetic Micro-Nano-Hybrid Structured TiO_2_ Overcomes the Biological Dilemma of Osteoblasts. Dent. Mater..

[19] Hori N., Ueno T., Suzuki T., Yamada M., Att W., Okada S., Ohno A., Aita H., Kimoto K., Ogawa T. (2010). Ultraviolet Light Treatment for the Restoration of Age-Related Degradation of Titanium Bioactivity. Int. J. Oral Maxillofac. Implant..

[20] Iwasa F., Tsukimura N., Sugita Y., Kanuru R.K., Kubo K., Hasnain H., Att W., Ogawa T. (2011). TiO_2_ Micro-Nano-Hybrid Surface to Alleviate Biological Aging of UV-Photofunctionalized Titanium. Int. J. Nanomed..

[21] Minamikawa H., Att W., Ikeda T., Hirota M., Ogawa T. (2016). Long-Term Progressive Degradation of the Biological Capability of Titanium. Materials.

[22] Suzuki T., Hori N., Att W., Kubo K., Iwasa F., Ueno T., Maeda H., Ogawa T. (2009). Ultraviolet Treatment Overcomes Time-Related Degrading Bioactivity of Titanium. Tissue Eng. Part A.

[23] Att W., Ogawa T. (2012). Biological Aging of Implant Surfaces and Their Restoration with Ultraviolet Light Treatment: A Novel Understanding of Osseointegration. Int. J. Oral Maxillofac. Implant..

[24] Ogawa T. (2012). UV-Photofunctionalization of Titanium Implants. Oral Craniofac. Tissue Eng..

[25] Att W., Hori N., Iwasa F., Yamada M., Ueno T., Ogawa T. (2009). The Effect of UV-Photofunctionalization on the Time-Related Bioactivity of Titanium and Chromium-Cobalt Alloys. Biomaterials.

[26] Att W., Hori N., Takeuchi M., Ouyang J., Yang Y., Anpo M., Ogawa T. (2009). Time-Dependent Degradation of Titanium Osteoconductivity: An Implication of Biological Aging of Implant Materials. Biomaterials.

[27] Suzuki T., Kubo K., Hori N., Yamada M., Kojima N., Sugita Y., Maeda H., Ogawa T. (2010). Nonvolatile Buffer Coating of Titanium to Prevent Its Biological Aging and for Drug Delivery. Biomaterials.

[28] Hori N., Ueno T., Minamikawa H., Iwasa F., Yoshino F., Kimoto K., Lee M.C., Ogawa T. (2010). Electrostatic Control of Protein Adsorption on UV-Photofunctionalized Titanium. Acta Biomater..

[29] Lee J.H., Ogawa T. (2012). The Biological Aging of Titanium Implants. Implant Dent..

[30] Ghassemi A., Ishijima M., Hasegawa M., Mohammadzadeh Rezaei N., Nakhaei K., Sekiya T., Torii Y., Hirota M., Park W., Miley D.D. (2018). Biological and Physicochemical Characteristics of 2 Different Hydrophilic Surfaces Created by Saline-Storage and Ultraviolet Treatment. Implant Dent..

[31] Aita H., Att W., Ueno T., Yamada M., Hori N., Iwasa F., Tsukimura N., Ogawa T. (2009). Ultraviolet Light-Mediated Photofunctionalization of Titanium to Promote Human Mesenchymal Stem Cell Migration, Attachment, Proliferation and Differentiation. Acta Biomater..

[32] Aita H., Hori N., Takeuchi M., Suzuki T., Yamada M., Anpo M., Ogawa T. (2009). The Effect of Ultraviolet Functionalization of Titanium on Integration with Bone. Biomaterials.

[33] Minamikawa H., Ikeda T., Att W., Hagiwara Y., Hirota M., Tabuchi M., Aita H., Park W., Ogawa T. (2014). Photofunctionalization Increases the Bioactivity and Osteoconductivity of the Titanium Alloy Ti6Al4V. J. Biomed. Mater. Res. A.

[34] Saita M., Ikeda T., Yamada M., Kimoto K., Lee M.C., Ogawa T. (2016). UV Photofunctionalization Promotes Nano-Biomimetic Apatite Deposition on Titanium. Int. J. Nanomed..

[35] Tsukimura N., Yamada M., Iwasa F., Minamikawa H., Att W., Ueno T., Saruwatari L., Aita H., Chiou W.A., Ogawa T. (2011). Synergistic Effects of UV Photofunctionalization and Micro-Nano Hybrid Topography on the Biological Properties of Titanium. Biomaterials.

[36] Tabuchi M., Hamajima K., Tanaka M., Sekiya T., Hirota M., Ogawa T. (2021). UV Light-Generated Superhydrophilicity of a Titanium Surface Enhances the Transfer, Diffusion and Adsorption of Osteogenic Factors from a Collagen Sponge. Int. J. Mol. Sci..

[37] Aita H., Oh W., Kubo K., Tsukimura N., Maeda H., Ogawa T. (2008). Light-Induced Bone Cement-Philic Titanium Surface. J. Mater. Sci..

[38] Okubo T., Ikeda T., Saruta J., Tsukimura N., Hirota M., Ogawa T. (2020). Compromised Epithelial Cell Attachment After Polishing Titanium Surface and Its Restoration by UV Treatment. Materials.

[39] Okubo T., Tsukimura N., Taniyama T., Ishijima M., Nakhaei K., Rezaei N.M., Hirota M., Park W., Akita D., Tateno A. (2018). Ultraviolet Treatment Restores Bioactivity of Titanium Mesh Plate Degraded by Contact with Medical Gloves. J. Oral Sci..

[40] Nakhaei K., Ishijima M., Ikeda T., Ghassemi A., Saruta J., Ogawa T. (2020). Ultraviolet Light Treatment of Titanium Enhances Attachment, Adhesion, and Retention of Human Oral Epithelial Cells via Decarbonization. Materials.

[41] Hirota M., Ikeda T., Tabuchi M., Iwai T., Tohnai I., Ogawa T. (2014). Effect of Ultraviolet-Mediated Photofunctionalization for Bone Formation Around Medical Titanium Mesh. J. Oral Maxillofac. Surg..

[42] Ikeda T., Hagiwara Y., Hirota M., Tabuchi M., Yamada M., Sugita Y., Ogawa T. (2014). Effect of Photofunctionalization on Fluoride-Treated Nanofeatured Titanium. J. Biomater. Appl..

[43] Tabuchi M., Ikeda T., Hirota M., Nakagawa K., Park W., Miyazawa K., Goto S., Ogawa T. (2015). Effect of UV Photofunctionalization on Biologic and Anchoring Capability of Orthodontic Miniscrews. Int. J. Oral Maxillofac. Implant..

[44] Pyo S.W., Park Y.B., Moon H.S., Lee J.H., Ogawa T. (2013). Photofunctionalization Enhances Bone-Implant Contact, Dynamics of Interfacial Osteogenesis, Marginal Bone Seal, and Removal Torque Value of Implants: A Dog Jawbone Study. Implant Dent..

[45] Funato A., Ogawa T. (2013). Photofunctionalized Dental Implants: A Case Series in Compromised Bone. Int. J. Oral Maxillofac. Implant..

[46] Hirota M., Ikeda T., Tabuchi M., Nakagawa K., Park W., Ishijima M., Tsukimura N., Hagiwara Y., Ogawa T. (2016). Bone Generation Profiling Around Photofunctionalized Titanium Mesh. Int. J. Oral Maxillofac. Implant..

[47] Hirota M., Ikeda T., Tabuchi M., Ozawa T., Tohnai I., Ogawa T. (2017). Effects of Ultraviolet Photofunctionalization on Bone Augmentation and Integration Capabilities of Titanium Mesh and Implants. Int. J. Oral Maxillofac. Implant..

[48] Hirota M., Ozawa T., Iwai T., Mitsudo K., Ogawa T. (2020). UV-Mediated Photofunctionalization of Dental Implant: A Seven-Year Results of a Prospective Study. J. Clin. Med..

[49] Hirota M., Ozawa T., Iwai T., Ogawa T., Tohnai I. (2016). Implant Stability Development of Photofunctionalized Implants Placed in Regular and Complex Cases: A Case-Control Study. Int. J. Oral Maxillofac. Implant..

[50] Hori N., Iwasa F., Tsukimura N., Sugita Y., Ueno T., Kojima N., Ogawa T. (2011). Effects of UV Photofunctionalization on the Nanotopography Enhanced Initial Bioactivity of Titanium. Acta Biomater..

[51] Ishijima M., de Avila E.D., Nakhaei K., Shi W., Lux R., Ogawa T. (2019). Ultraviolet Light Treatment of Titanium Suppresses Human Oral Bacterial Attachment and Biofilm Formation: A Short-Term In Vitro Study. Int. J. Oral Maxillofac. Implant..

[52] Ishijima M., Ghassemi A., Soltanzadeh P., Tanaka M., Nakhaei K., Park W., Hirota M., Tsukimura N., Ogawa T. (2016). Effect of UV Photofunctionalization on Osseointegration in Aged Rats. Implant Dent..

[53] Iwasa F., Baba K., Ogawa T. (2016). Enhanced Intracellular Signaling Pathway in Osteoblasts on Ultraviolet Lighttreated Hydrophilic Titanium. Biomed. Res..

[54] Iwasa F., Hori N., Ueno T., Minamikawa H., Yamada M., Ogawa T. (2010). Enhancement of Osteoblast Adhesion to UV-Photofunctionalized Titanium via an Electrostatic Mechanism. Biomaterials.

[55] Iwasaki C., Hirota M., Tanaka M., Kitajima H., Tabuchi M., Ishijima M., Park W., Sugita Y., Miyazawa K., Goto S. (2020). Tuning of Titanium Microfiber Scaffold with UV-Photofunctionalization for Enhanced Osteoblast Affinity and Function. Int. J. Mol. Sci..

[56] Soltanzadeh P., Ghassemi A., Ishijima M., Tanaka M., Park W., Iwasaki C., Hirota M., Ogawa T. (2017). Success Rate and Strength of Osseointegration of Immediately Loaded UV-Photofunctionalized Implants in a Rat Model. J. Prosthet. Dent..

[57] Sugita Y., Honda Y., Kato I., Kubo K., Maeda H., Ogawa T. (2014). Role of Photofunctionalization in Mitigating Impaired Osseointegration Associated with type 2 Diabetes in Rats. Int. J. Oral Maxillofac. Implant..

[58] Taniyama T., Saruta J., Mohammadzadeh Rezaei N., Nakhaei K., Ghassemi A., Hirota M., Okubo T., Ikeda T., Sugita Y., Hasegawa M. (2020). UV-Photofunctionalization of Titanium Promotes Mechanical Anchorage in A Rat Osteoporosis Model. Int. J. Mol. Sci..

[59] Ueno T., Yamada M., Hori N., Suzuki T., Ogawa T. (2010). Effect of Ultraviolet Photoactivation of Titanium on Osseointegration in a Rat Model. Int. J. Oral Maxillofac. Implant..

[60] Ueno T., Yamada M., Suzuki T., Minamikawa H., Sato N., Hori N., Takeuchi K., Hattori M., Ogawa T. (2010). Enhancement of Bone-Titanium Integration Profile with UV-Photofunctionalized Titanium in a Gap Healing Model. Biomaterials.

[61] Ishikawa T., Vela X., Kida K., Moroi H., Kitajima H., Ogawa T. (2014). Restoration of Optimum Esthetics in Complex Clinical Situations Using an Interdisciplinary Strategy in Combination with Advanced Techniques and Technologies in Regenerative Medicine. J. Cosmet. Dent..

[62] Kitajima H., Ogawa T. (2016). The Use of Photofunctionalized Implants for Low or Extremely Low Primary Stability Cases. Int. J. Oral Maxillofac. Implant..

[63] Ueno T., Ikeda T., Tsukimura N., Ishijima M., Minamikawa H., Sugita Y., Yamada M., Wakabayashi N., Ogawa T. (2016). Novel Antioxidant Capability of Titanium Induced by UV Light Treatment. Biomaterials.

[64] Tsukimura N., Yamada M., Aita H., Hori N., Yoshino F., Chang-Il Lee M., Kimoto K., Jewett A., Ogawa T. (2009). N-Acetyl Cysteine (NAC)-Mediated Detoxification and Functionalization of Poly(Methyl Methacrylate) Bone Cement. Biomaterials.

[65] Nakamura H., Shim J., Butz F., Aita H., Gupta V., Ogawa T. (2006). Glycosaminoglycan Degradation Reduces Mineralized Tissue-Titanium Interfacial Strength. J. Biomed. Mater. Res. A.

[66] Ueno T., Yamada M., Igarashi Y., Ogawa T. (2011). N-Acetyl Cysteine Protects Osteoblastic Function from Oxidative Stress. J. Biomed. Mater. Res. A.

[67] Yamada M., Ueno T., Minamikawa H., Sato N., Iwasa F., Hori N., Ogawa T. (2010). N-Acetyl Cysteine Alleviates Cytotoxicity of Bone Substitute. J. Dent. Res..

[68] Aita H., Tsukimura N., Yamada M., Hori N., Kubo K., Sato N., Maeda H., Kimoto K., Ogawa T. (2010). N-Acetyl Cysteine Prevents Polymethyl Methacrylate Bone Cement Extract-Induced Cell Death and Functional Suppression of Rat Primary Osteoblasts. J. Biomed. Mater. Res. A.

[69] Tsukimura N., Ueno T., Iwasa F., Minamikawa H., Sugita Y., Ishizaki K., Ikeda T., Nakagawa K., Yamada M., Ogawa T. (2011). Bone Integration Capability of Alkali- and Heat-Treated Nanobimorphic Ti-15Mo-5Zr-3Al. Acta Biomater..

[70] Yamada M., Ueno T., Minamikawa H., Ikeda T., Nakagawa K., Ogawa T. (2013). Early-Stage Osseointegration Capability of a Submicrofeatured Titanium Surface Created by Microroughening and Anodic Oxidation. Clin. Oral Implant. Res..

[71] Kojima N., Ozawa S., Miyata Y., Hasegawa H., Tanaka Y., Ogawa T. (2008). High-Throughput Gene Expression Analysis in Bone Healing Around Titanium Implants by DNA Microarray. Clin. Oral Implant. Res..

[72] Rezaei N.M., Hasegawa M., Ishijima M., Nakhaei K., Okubo T., Taniyama T., Ghassemi A., Tahsili T., Park W., Hirota M. (2018). Biological and Osseointegration Capabilities of Hierarchically (Meso-/micro-/nanoscale) Roughened Zirconia. Int. J. Nanomed..

[73] Hasegawa M., Saruta J., Hirota M., Taniyama T., Sugita Y., Kubo K., Ishijima M., Ikeda T., Maeda H., Ogawa T. (2020). A Newly Created Meso-, Micro-, and Nano-Scale Rough Titanium Surface Promotes Bone-Implant Integration. Int. J. Mol. Sci..

[74] Kubo K., Tsukimura N., Iwasa F., Ueno T., Saruwatari L., Aita H., Chiou W.A., Ogawa T. (2009). Cellular Behavior on TiO_2_ Nanonodular Structures in a Micro-to-Nanoscale Hierarchy Model. Biomaterials.

[75] Ogawa T., Saruwatari L., Takeuchi K., Aita H., Ohno N. (2008). Ti Nano-Nodular Structuring for Bone Integration and Regeneration. J. Dent. Res..

[76] Ueno T., Tsukimura N., Yamada M., Ogawa T. (2011). Enhanced Bone-Integration Capability of Alkali- and Heat-Treated Nanopolymorphic Titanium in Micro-to-Nanoscale Hierarchy. Biomaterials.

[77] Yamada M., Ueno T., Tsukimura N., Ikeda T., Nakagawa K., Hori N., Suzuki T., Ogawa T. (2012). Bone Integration Capability of Nanopolymorphic Crystalline Hydroxyapatite Coated on Titanium Implants. Int. J. Nanomed..

[78] Uno M., Hayashi M., Ozawa R., Saruta J., Ishigami H., Ogawa T. (2020). Mechanical Interlocking Capacity of Titanium with Respect to Surface Morphology and Topographical Parameters. J. Dent. Oral Biol..

[79] Uno M., Ozawa R., Hamajima K., Saruta J., Ishigami H., Ogawa T. (2020). Variation in Osteoblast Retention Ability of Titanium Surfaces with Different Topographies. J. Dent. Oral Biol..

[80] Butz F., Ogawa T., Nishimura I. (2011). Interfacial Shear Strength of Endosseous Implants. Int. J. Oral Maxillofac. Implant..

[81] Ozawa S., Ogawa T., Iida K., Sukotjo C., Hasegawa H., Nishimura R.D., Nishimura I. (2002). Ovariectomy Hinders the Early Stage of Bone-Implant Integration: Histomorphometric, Biomechanical, and Molecular Analyses. Bone.

[82] Saruta J., Sato N., Ishijima M., Okubo T., Hirota M., Ogawa T. (2019). Disproportionate Effect of Sub-Micron Topography on Osteoconductive Capability of Titanium. Int. J. Mol. Sci..

[83] Sato N., Kubo K., Yamada M., Hori N., Suzuki T., Maeda H., Ogawa T. (2009). Osteoblast Mechanoresponses on Ti with Different Surface Topographies. J. Dent. Res..

[84] Noda M., Rodan G.A. (1987). Type beta transforming growth factor (TGF beta) regulation of alkaline phosphatase expression and other phenotype-related mRNAs in osteoblastic rat osteosarcoma cells. J. Cell. Physiol..

[85] Elias J.A., Tanq W., Horowitz M.C. (1995). Cytokine and hormonal stimulation of human osteosarcoma interleukin-11 production. Endcrinology.

[86] Yashiro R., Nagasawa T., Kiji M., Hormdee D., Kobayashi H., Koshy G., Nitta H., Ishikawa I. (2006). Transforming growth factor-beta stimulates interleukin-11 production by human periodontal ligament and gingival fibroblasts. J. Clin. Periodontol..

[87] De Avila E.D., Lima B.P., Sekiya T., Torii Y., Ogawa T., Shi W., Lux R. (2015). Effect of UV-photofunctionalization on oral bacterial attachment and biofilm formation to titanium implant material. Biomaterials.

[88] Shokeen B., Zamani L., Zadmehr S., Pouraghaie S., Ozawa R., Yilmaz B. (2021). Surface characterization and assessment of biofilm formation on two titanium-based implant coating materials. Front. Dent. Med..

[89] Schlegel K.A., Prechtl C., Möst T., Seidl C., Lutz R., von Wilmowsky C. (2013). Osseointegration of SLActive Implants in Diabetic Pigs. Clin. Oral Implant. Res..

[90] Zinelis S., Silikas N., Thomas A., Syres K., Eliades G. (2012). Surface Characterization of SLActive Dental Implants. Eur. J. Esthet. Dent..

[91] Anselme K. (2000). Osteoblast Adhesion on Biomaterials. Biomaterials.

[92] Henningsen A., Smeets R., Hartjen P., Heinrich O., Heuberger R., Heiland M., Precht C., Cacaci C. (2018). Photofunctionalization and Non-Thermal Plasma Activation of Titanium Surfaces. Clin. Oral Investig..

[93] International Organization for Standardization (1975). ISO 2533:1975 Standard Atmosphere.

